# Shortening of the Burnout Assessment Tool (BAT)—from 23 to 12 items using content and Rasch analysis

**DOI:** 10.1186/s12889-022-12946-y

**Published:** 2022-03-22

**Authors:** Emina Hadžibajramović, Wilmar Schaufeli, Hans De Witte

**Affiliations:** 1 Institute of Stress Medicine, Region Västra Götaland, Carl Skottsbergs gata 22 B, 413 19 Göteborg, Sweden; 2 School of Public Health and Community Medicine, Institute of Medicine, Sahlgrenska Academy, University of Gothenburg, Gothenburg, Sweden; 3 Department of Psychology, Utrecht University, Utrecht, The Netherlands; 4 Research Unit Occupational & Organizational Psychology and Professional Learning, Leuven, KU Belgium; 5 Optentia Research Unit, North-West University, Potchefstroom, South Africa

**Keywords:** Burnout, BAT, Psychometrics, Validation

## Abstract

**Background:**

Burnout is related to huge costs, for both individuals and organizations and is recognized as an occupational disease or work-related disorder in many European countries. Given that burnout is a major problem it is important to measure the levels of burnout in a valid and reliable way.

The Burnout Assessment Tool (BAT) is a newly developed self-report questionnaire to measure burnout. So far, studies concerning the psychometric properties of the original version of the Burnout Assessment Tool (BAT) including 23 items show promising results and suggest that the instrument can be used in many different settings.

For various reasons there is a need of a shorter instrument. For example, burnout questionnaires are typically included in employee surveys to evaluate psychosocial risk-factors, which according to the European Occupational Safety and Health Framework Directive, should be carried out in organizations on a regular basis. The aims of this paper are to develop a shorter version of the BAT, including only 12 items (BAT12) and to evaluate its construct validity and differential item functioning regarding age, gender and country.

**Methods:**

Using data from representative samples of working populations in the Netherlands and Belgium (Flanders) a shorter version of the BAT was developed by combining quantitative (Rasch analysis) and qualitative approaches (item content analysis and expert judgements). Construct validity of the new BAT12 was evaluated by means of Rasch analysis.

**Results:**

In an iterative procedure, deleting one item from each subscale at each step, a short version of the BAT – BAT12 was developed. The BAT12 fulfils the measurement criteria according to the Rasch model after accounting for local dependency between items within each subscale. The four subscales can be combined into a single burnout score.

**Conclusion:**

The new BAT12 developed in the present study maintains the breath of item content of the original version of the BAT. The new BAT12 has sound psychometric properties. The scale works invariantly for older and younger, women and men and across two countries. A shorter version of the BAT is timesaving compared to the BAT23 and can be used in e.g. employee surveys.

**Supplementary Information:**

The online version contains supplementary material available at 10.1186/s12889-022-12946-y.

## Background

Burnout is a metaphor that refers to the loss of mental energy, or more specifically to a work-related state of mental exhaustion [1, 2]. Since its introduction, at the end of the 1970s it has been studied extensively and a recent overview showed convincingly that burnout is related to huge costs, both for the individual as well as organizations [3]. For instance, burnout is associated with poor physical and mental health of employees such as, cardio-vascular disease, anxiety and depression, musculoskeletal disorders, insomnia, and psychosomatic complaints. For organizations, burnout leads to high replacement costs due to turnover, sickness absence and work incapacitation, but also to poor business outcomes in terms of job performance, occupational safety, service quality, and productivity. Not surprisingly, burnout is recognized as an occupational disease or work-related disorder in many European countries, including Belgium and the Netherlands [4]. According to the European Occupational Safety and Health Framework Directive (89/391/EEC-OSH) employers must evaluate all the risks to the safety and health of their workers. In order to comply with this directive a valid and reliable instrument should be available that can be used to measure employee’s burnout levels.

To date, the Maslach Burnout Inventory (MBI) [5] is almost universally used to measure burnout. It is estimated that in 88% of all scientific papers on burnout, the MBI is the instrument of choice [6]. The MBI builds on the definition of burnout as a syndrome of emotional exhaustion, depersonalization and reduced personal accomplishment [7], later denoted as exhaustion, cynicism and lack of professional efficacy, respectively [8].

In fact, because of the dominance of the MBI, burnout is what the MBI measures, and vice versa. Needless to say, that this circularity of concept and assessment is undesirable because it hinders fresh and innovative research that increases our understanding of burnout. Moreover, the MBI has been developed as a research instrument and not as a tool to assess levels of burnout in organizations. Even more importantly, over the years a number of conceptual as well as technical concerns have been raised against the use of the MBI. For instance, it was argued that rather than a constituting element, poor professional efficacy should be considered a consequence of burnout [2]. Conversely, it was maintained that impaired cognitive functioning is wrongly not taken into account as an indicator of burnout in the MBI [9]. Psychometrically speaking the MBI has been criticized – amongst others – for: (1) skewed answering patterns that may affect its reliability; (2) reversing positively worded items for evaluating a negative psychological state; (3) producing three different subscale scores instead of a single, composite burnout score [10].

Hence, recently an alternative self-report questionnaire has been developed that effectively addresses these conceptual and technical issues; the Burnout Assessment Tool (BAT) [10] (see also, www.burnoutassessmenttool.be). The BAT includes four subscales: exhaustion (i.e., extreme tiredness); mental distance (i.e., psychological withdrawal); cognitive and emotional impairment (i.e., reduced ability to regulate one’s cognitive and emotional processes, respectively). So far, the psychometric features of the BAT seem encouraging. For instance, using nationally representative samples of seven different countries, De Beer et al. [11] showed that the postulated second-order factor structure with all four BAT subscales loading on one general, composite score was invariant across countries. In a similar vein, a rigorous Rasch analysis attested the one-dimensionality of the BAT [12], suggesting that a single score can be used to assess the employee’s level of burnout. These results confirm that, contrary to the MBI, the BAT conceives burnout as a syndrome that consists of set of related symptoms that refer to one underlying psychological condition. Furthermore, multitrait-multimethod analyses showed convergent and discriminant validity of the BAT vis-à-vis other burnout measures, such as the MBI [10, 13]. That is, although the BAT partially overlaps with other burnout instruments, its distinctiveness was established as well. Finally, results suggest that burnout as assessed by the BAT can be discriminated from other, related aspects of employee well-being such as work engagement, job boredom and workaholism [10, 13].

Typically, burnout questionnaires are included in employee surveys to evaluate psychosocial risk-factors, which according to the European Occupational Safety and Health Framework Directive should be carried out in organizations on a regular basis. Because employee surveys tend to be rather long and employers usually impose time constraints for surveying employees during their work time, there is increasing pressure on researchers to develop valid, reliable, yet short measures without redundant items [14]. Such concise measures also reduce participant’s fatigue, frustration, and the likelihood of refusing to participate because the survey is perceived to be too long and time consuming [15].

Shorter instruments are requested for both academic research purposes and for practical purposes such as employee surveys as they may be more efficient from a practical point of view. Construction and use of short scales bring together theoretical, statistical, and practical perspectives. Removing items from psychometrically sound instruments goes against the traditional psychometric view that many items are needed for reliable and valid measurement [16] and might result in lower reliability and poorer construct coverage. With the growing need and use of short questionnaires, there is also a growing literature regarding scale-shortening strategies and their consequences [17–27]. The specific number of items included or required in a questionnaire is of course contextual and depends on the complexity of the construct of interest. Therefore, general recommendations regarding the number of items in a questionnaire are perhaps not always useful.

Different strategies for scale-shortening and choosing the number of items, are available and include both quantitative (data-driven) and qualitative (theoretical driven) strategies. One data-driven strategy is to select items that maximize the internal consistency coefficient, which sometime might be associated with the risk of narrowing the construct coverage. The Rasch analysis is another strategy. If a set of items fits a Rasch model, then any subset of these items would also fit the model. As a consequence, the selection of the items can be very easy, given that there is no local dependency and/or differential item functioning. In a previous study [12] we have shown that the BAT23 fits the Rasch model, after accounting for local dependency by combining the items within each subscale into four testlets. However, due to local dependency it was not possible to choose any subset of items out of the original 23 items.

In order to shorten the BAT, we used a combination of a quantitative (Rasch analysis) as well as qualitative approach (item content analysis and expert judgement). The Rasch measurement model, usually referred to as Rasch analysis, belongs to the item response theory or modern test theory as opposed to the classical test theory. The Rasch analysis can be used for shortening scales as it provides information about each item in several ways and in that way helps in selecting items that improve the short scale’s accuracy [17, 18, 28]. Besides a statistics-driven strategy, a qualitative analysis based on item content analysis was employed as well as judgmental strategy. This will guide the selection of items on the basis of expert judgment on which items best cover the construct of interest, and with the goal to preserve the same content validity as in the original version of the BAT.

An equal number of items in each subscale is recommended when a questionnaire is used as self-assessment and the respondent is also scoring and interpreting the test results on its own [28]. In that way the scoring of the test would be less complicated and more transparent to the test user. To fulfil these requirements, the minimum number of items within each subscale was set to three. The choice of three items was a matter of balance as three out of the four BAT subscales consist of five items each. Reducing to four instead of five items is not really time saving for the respondents and two items per subscale are perhaps not enough to ensure appropriate construct coverage within each subscale.

The current paper has four aims: (1) to shorten the original 23-item version of the BAT to a version that includes only 12 items, 3 items for each subscale, using a combination of content and Rasch analysis; (2) to evaluate construct validity of the BAT12 using Rasch analysis; (3) to evaluate whether the items of the BAT12—like those of the BAT23—can be combined into a single burnout score; (4) to evaluate possible differential item functioning of the BAT12 regarding gender, age and country.

## Methods

### Study design and population

Analysis was done using the original Dutch version of the BAT23 and data from two representative samples of the working populations in terms of age, gender and industry in the Netherlands and Flanders, Belgium, respectively (*n* = 1500 from each country). Prior to filling out the questionnaire, all participants were informed about the purpose of the study, that participation was voluntary, that they could stop at any moment if they wished to do so, that questions could be directed to a contact person (name and email address provided) and that complaints could be filed with the ethical committee (email address provided). Moreover, participants declared that they agreed with these terms by clicking on “next”. This study was approved by the ethical committee Sociaal Maatschappelijke Ethische Commissie (Social and Societal Ethics Committee (SMEC) of KU Leuven) (https://www.kuleuven.be/english/research/ethics/committees/smec) on June 16, 2016 (reference number: G-2016 06 2027). All the methods were in accordance with the declaration of Helsinki or in accordance with relevant national/institutional guidelines.

Details about the sampling procedure and sample characteristics are described in previous studies [10, 12] as well as in the BAT test-manual [29]. Complete cases on all items were obtained for *n* = 2978 (NL = 1500, FL = 1478) and these were considered for analyses.

In this study differential item functioning (DIF) was evaluated for age, gender and country. Given the same level of the latent trait (i.e. burnout), the scale should function identically for all comparable groups. In the presence of DIF, comparable groups (e.g., women and men) score differently on a specific item, even though they have the similar levels of burnout. An equal number of cases within each of the compared groups is recommended to ensure that if there was DIF, one group does not dominate in the estimates of parameters [30]. Given that the data material was large, it was possible to adapt a cross-validation strategy (i.e. analyze data twice using two random subsamples) to check the robustness of the results. Therefore, the total sample was divided into 4 homogenous strata of men/NL, men/FL, women/NL and women/FL. Then a random sample of 200 respondents from each stratum was drawn twice, resulting in two subsamples of 800 individuals. Age was divided by the median. Median age in the two samples was 41.

### The burnout assessment tool (BAT)

The Burnout Assessment Tool (BAT) is a self-report a questionnaire consisting of 23 items (see Additional file 1) that includes four dimensions: exhaustion (EX; 8 items), mental distance (MD; 5 items), cognitive impairment (CI; 5 items) and emotional impairment (EI; 5 items). All items are expressed as statements with five frequency-based response categories (1 = never, 2 = rarely, 3 = sometimes, 4 = often, 5 = always). Detailed information about the development of the BAT is provided elsewhere [10] as well as in the BAT test-manual [29]. A previous study showed that the BAT23 has sound psychometric properties and satisfies the requirements for the measurement criteria according to the Rasch model when subscales are used instead of the individual items [12]. The BAT score also works invariantly for women and men, younger and older respondents, and across both countries.

### Data analysis

The shortening of the scale was done by combining quantitative (statistics-driven) and qualitative strategies.

The qualitative strategy incorporated item content analysis, also known as a subject matter analysis [31], as well as expert judgement strategy. Subject matter analysis classifies items into five categories: (1) no problems with the item, (2) wording errors, (3) wording similar to one or more other items, (4) item measures the same characteristic as one or more other items, and (5) item is an unclear measure of the construct. All items were analyzed by the first author. Then all authors read and commented the results and adjustments were made until all authors agreed on the classifications for all items.

The statistical part of the analysis was based on Rasch analysis. Short introductions to Rasch analysis can be found elsewhere [32–34] and a comprehensive overview of the statistical theory of Rasch models is presented in a textbook [35]. The goal of the Rasch analysis is to evaluate whether the observed data satisfy the assumptions of the Rasch model, in which case the questionnaire has solid psychometric properties. The advantage of the Rasch model over classical test theory approaches such as factor analysis, is that the normal score distribution of items is not required.

The following four item fit indicators were examined: (1) the item’s ability to discriminate (based on item fit residuals expected to range between ± 2.5 and χ2 statistic); (2) appropriateness of the response categories (threshold ordering); (3) response independence relative to other items (residual correlations); (4) and the absence of differential item functioning (DIF) for age, gender and country. Absence of DIF means that given the same level of burnout, items should function similarly for all comparable groups (women and men, older and younger age, NL and FL). Any residual correlation between the items 0.2 above the average observed correlation is indicative of response dependency [36].

Besides item fit, the overall fit to the Rasch model was evaluated at each step by means of summary fit statistics: the item-trait interaction statistic (non-significant χ^2^ statistic), and person and item fit residuals (expected values around zero mean and SD of 1). The internal consistency and the power of the scale to discriminate among respondents were evaluated with the Person Separation Index (PSI). The PSI is similar to Cronbach’s alpha with a range of 0 to 1. Dimensionality of the scale was tested by Smith’s test of unidimensionality [37]. For this test, first a principal component analysis (PCA) on residuals is performed. Next, items loading positively and negatively on the first principal component are used to obtain an independent person estimate. In the next step, independent t-tests for differences among these estimates for each person were performed [37]. Less than 5% of such tests being outside the range of ± 1.96 support the unidimensionality of the scale. A 95% binomial confidence interval of proportions [38] was used to show that the lower limit of the observed proportion is below the 5% level [37].

Analyses were performed independently on the two random samples, each consisting of 800 participants. A sample size of 800 is sufficient to yield a high degree of precision [39]. All analyses were done in RUMM2030 [40]. The item’s fit was analyzed using the partial credit model for polytomous cases [41]. To control for the large number of comparisons, the significance level was set at 0.01 and Bonferroni adjusted.

When local dependency was detected, a method of combining correlated items into testlets was applied as suggested by Marais and colleagues [42–44]. According to this method, correlated items are combined into one or more testlets (preferably based on theoretical reasoning) and the data are re-analyzed using testlets instead of individual items. The testlets’ model fit was compared with the fit obtained from the BAT12 analysis. The latent correlation among the subscales was also calculated, as well as the proportion of the non-error common variance accounted for when the testlets were added to constitute a total score (also known as explained common variance) [42, 45, 46].

DIF was evaluated using ANOVA on standardized residuals which enables separate estimations of misfit along the latent trait, uniform and non-uniform DIF. Uniform DIF implies a systematic difference in the response to an item that is consistent across the entire range of the latent trait of burnout. Non-uniform DIF means an interaction and implies that the magnitude of DIF is not consistent across the continuum of latent trait. The distinction between real and artificial DIF, and the magnitude and the impact of DIF were investigated by the methods recommended by Andrich and Hagquist [30, 47, 48]. In addition to formal tests, DIF was also evaluated graphically by means of the item characteristic curve.

Targeting (distribution of the persons and the items on a common logit scale) was evaluated visually by the person-item-threshold graph. Targeting is an aspect of how well the BAT12 items are targeted for severity levels of burnout as reported by the respondents. This is important for the precision of the person estimates. In case of good fit to the Rasch model, person estimates from the Rasch analysis, which are logits, can be transformed into a convenient range (in this case values in the range 1 to 5), henceforth referred to as metric score [49].

### The shortening procedure

Items were eliminated iteratively, one at a time from each subscale, re-running the Rasch analysis after each step and comparing summary fit statistics. The first analysis included all 23 items (BAT23). Thereafter, one item from each subscale was eliminated at each step and the process was repeated until three items remained in the subscales MD, CI and EI and six items remained in the EX- subscale (BAT19 and BAT15). The procedure then continued by eliminating one item from the EX-subscale at each step until three items remained in this subscale as well (BAT14, BAT13 and BAT12). As a result, the final model (BAT12) included the same number of items within each subscale. Lastly, the BAT12 was fitted to the Rasch model and evaluated using summary and item fit statistics.

Items that were judged to perform poorly based on one or more of the item fit indicators and/or results of the subject matter analysis were selected as candidates for elimination at each step. In cases when the decision about item elimination could not be reached based on item fit statistics and/or subject matter analysis, two additional criteria were used. One criterion was to investigate item and threshold locations (positionings) on a latent trait in order to maximize the spread of the items across the latent burnout continuum. Thresholds partition the latent continuum of each item into ordered categories and are the points between any two adjacent categories in which the conditional probability of either response is equally likely. The other criterion was to evaluate the meaning and the content of the item and keep items that were judged important theoretically, to ensure proper content validity of each subscale.

## Results, sample 1

### Subject matter analysis

All BAT items were judged using categories (1) to (5) described above. The BAT is a newly developed questionnaire and the conceptualization, construction as well as the items formulation were described in detail in a recently published paper [10] and in the BAT manual [29]. All items were considered as relevant for measuring the burnout construct and none of the 23 items were classified into category (5; ‘item is an unclear measure of the construct’). Furthermore, none of the items were classified into category (3; ‘wording similar to one or more other items’).

#### Exhaustion

Six of the eight items (items EX2 through EX7) were categorized as *(1*) ‘no problems with the item’*.* The contents of item EX1 (*At work, I feel mentally exhausted*) and item EX8 (*At the end of my working day, I feel mentally exhausted and drained*) are not identical, but partly overlap as both items address aspects of mental exhaustion. They were therefore *classified as (4) ‘item measures same characteristic as one or more other items’.*

#### Mental distance

Items MD1 (*I struggle to find any enthusiasm for my work*) and MD4 (*I feel indifferent about my job*) could be classified as (4) *–* items that measure the same characteristic –but in opposite directions, expressed in positive (*enthusiasm*) and negative (*indifferent*) terms, respectively. On the other hand, one could also argue that these are actually different aspects of mental distance and thus the items could alternatively be classified as (1) no problem with the item. The remaining items MD2, MD3 and MD5 were classified as (1) ‘no problems with the item’.

#### Cognitive impairment

Items CI2, CI3 and CI5 were classified (1) ‘no problems with the item’. Items CI1 (*At work, I have trouble staying focused*) and CI4 (*When I’m working, I have trouble concentrating*) could be classified (4) ‘item measures same characteristic as one or more other items’.

#### Emotional impairment

Items EI2 through EI4 were classified as (1) ‘no problems with the item’. Items EI1 (*At work, I feel unable to control my emotions*) and EI5 (*At work, I may overact unintentionally*) were classified as related but also different and therefore both classified as (1) ‘no problems with the item’. EI1 is more general than EI5 as it includes not only one’s behavior (i.e., acting) but also one’s feeling, which not necessarily need to be acted out.

### The Rasch analysis

An initial Rasch analysis included all 23 items (BAT23). In the next two steps (analyses BAT19 and BAT15), four items were deleted at each step (one item from each subscale). At this stage of the analysis, the mental distance, and the cognitive and emotional impairment subscales were reduced to three items each, while the exhaustion subscale still had six items. Thus, the next goal was to further reduce the number of exhaustion items from six to three, deleting one item at each step (analyses BAT14, BAT13 and BAT12).

Overall fit statistics for each step are shown in Table 1. As seen in the Table 1, a deviation from the Rasch model was observed in the BAT23 analysis with a significant χ2 statistic. The values of item and person residuals means and SDs were higher than the expected value of 0 and 1 respectively. The PSI values was high (0.95) and the Smith’s test indicated problems with unidimensionality.

**Table 1:** Summary fit statistics, subsample 1 (*n* = 800)

	**Item residual**		**Person residual**		**Chi square**		**Unidimensionality**	
**Analysis name**	**Mean**	**SD**	**Mean**	**SD**	**Value**	**p**	**PSI**	**Test % (95% CI)**
BAT 23 items*	-0.15	2.91	-0.86	2.86	416.51	< 0.0001	0.95	20.9 (18.2;23.9)
BAT19	-0.12	2.55	-0.85	2.46	314.34	< 0.0001	0.94	15.8 (13.4;18.6)
BAT15a	-0.12	2.32	-0.80	2.26	192.35	0.0001	0.93	13.4 (11.2;16.4)
BAT15b	-0.19	2.25	-0.79	2.23	205.71	< 0.0001	0.93	13.8 (11.5;16.0)
BAT14	-0.10	2.21	-0.78	2.17	170.67	0.005	0.92	13.0 (10.8;15.6)
BAT13	-0.15	2.26	-0.80	2.18	156.73	0.008	0.91	12.1 (10.0;14.7)
BAT12	-0.19	2.32	-0.78	2.07	159.74	0.0009	0.91	13.0 (10.8;15.6)
BAT12 4 testlets	0.27	1.28	-0.51	1.14	45.06	0.14	0.82	4.4 (3.1;6.2)

^a^The BAT23 results are previously published in Hadžibajramović, E., W. Schaufeli, and H. De Witte, *A Rasch analysis of the Burnout Assessment Tool (BAT).* PLOS ONE, 2020. 15(11): p. e0242241

Compared to the first analysis, the value of the χ2 statistic dropped somewhat in the BAT19 analysis, but it was still high and significant. The mean and SD of the person and item fit residuals changed only marginally as well as the value of PSI, compared to the initial BAT23 analysis. The percentage of significant t-tests used for Smith’s test of unidimensionality decreased from 21 to 17 but is still very high compared to the expected value of 5, given local independence. The value of the χ2 statistic decreased with every step. For BAT13 and BAT12 the p-value approached the cut-off value of 0.01, so the fit to the model improved considerably. As expected, with decreasing the number of items, the PSI values also decreased at each step.

As mentioned above, four item fit indicators were evaluated at each step and used as criteria for deletions of items (i.e., threshold ordering, item fit residuals, residual correlations between items and DIF). All item fit statistics for the BAT23 analysis were published in a previous study [12] and therefore not repeated here. Item fit residuals for analyses BAT19 through BAT12 are shown in Table 2. The complete residual correlation tables for each analysis can be found in Additional file 2. DIF tables for age, gender and country are found in Additional file 3. The results for each subscale are presented below. In summary, item fit statistics identified several items with item fit residuals outside the predefined range of ± 2.5, high residual correlations between items within each subscale and some DIF issues. High residual correlations between item pairs belonging to different subscales were not observed in *any* of the analyses, indicating that item overlap is not an issue. All items had ordered thresholds in *all* analyses (BAT23, BAT19, BAT15, BAT14, BAT13, BAT12).

**Table 2:** Item fit residuals, subsample 1 (*n* = 800). Bold indicates significant item chi square (Bonferroni adjusted)

**Item**	**BAT19**	**BAT15a**	**BAT15b**	**BAT14**	**BAT13**	**BAT12**
EX1	-1.05	-0.96	-1.07	-0.99	-0.75	-0.64
EX2	-3.60	-3.20	-3.35			
EX3	2.93	2.00	1.84	1.98	2.90	3.03
EX4	1.19	1.47	1.39	1.53	2.04	2.42
EX5	1.46	1.29	1.11	1.32		
EX6	-2.76	-2.35	-2.30	-2.44	-1.88	
EX7	**4.42**					
EX8						
MD1	-3.61	-3.03	-3.18	-3.19	-2.99	-2.92
MD2						
MD3	-3.61	-3.34	-3.20	-3.40	-3.3	-3.43
MD4	1.25					
MD5	1.15	2.43	2.12	2.40	2.40	2.09
CI1	-0.77	0.39	0.20	0.11	0.08	-0.20
CI2						
CI3	-0.16					
CI4	.1.66	-0.72	-0.93	-0.94	-0.95	-1.03
CI5	-0.94	-0.77	0.37	0.46	0.32	0.08
EI1	-1.07	-0.48	0.03	-1.04	-1.52	-2.12
EI2	-1.48	-0.84	-0.49	-1.37	-1.91	-2.47
EI3	3.53		**4.60**			
EI4						
EI5	3.51	4.79		4.14	3.53	2.88

### The shortening of the BAT

#### Exhaustion

Among the eight exhaustion items, item fit residuals outside the predefined range of ± 2.5 were observed for items EX2, EX7 and EX8 (BAT23 analysis). DIF for age and gender was observed for item EX8. None of the items showed DIF between the two countries. Residual correlations indicated high values between several exhaustion items. Item EX8 was chosen as candidate for elimination due to misfit according to the item fit statistic, the high residual correlation with other items and DIF issues. According to the item content analysis, items EX8 and EX1 were classified into (4) ‘the item measures the same characteristic as on or more other items’. Tellingly, these items also had the highest observed residual correlations. Since both analytical strategies were pointing at the same item, the decision was made to eliminate item EX8.

In the next step (BAT19 analysis) items EX2, EX6 and E7 had problems with item fit residuals. For item EX7 misfit due to the item fit residual was also statistically significant. No DIF issues were found. The critical value for residual correlations in this analysis was 0.15, and several pairs of items exceeded that value: EX1-EX3 0.16, EX1-EX4 0.23, EX3-EX4 0.17, EX3-EX5 0.20, EX4-EX7 0.18. The decision was to discard EX7 (*When I exert myself at work, I get tired quicker than normal*) in the next step.

In the BAT15a analysis, only item EX2 had a high item fit residual -3.2 (Table 2). Residual correlations above the critical value (in the analysis > 0.13) were found for item pairs EX1-EX4 0.19, EX3-EX5 0.17 and EX3-EX4 0.14. Although the correlation between EX1 and EX4 was somewhat higher than the critical value, both items were kept in the analysis after item content evaluation, because they covered the mental and physical aspects of exhaustion, respectively (EX1: *At work, I feel mentally exhausted* and EX4: *At work, I feel physically exhausted*). Items EX3 (*After a day at work, I find it hard to recover my energy*) and EX5 (*When I get up in the morning, I lack the energy to start a new day at work*) had the lowest locations and are kept for that reason (Fig. 3). Thus, the decision here was to eliminate EX2. Evaluating the content of item EX2 (*Everything I do at work requires a great deal of effort*) strengthened the decision because it was judged that this aspect does not necessarily need to imply exhaustion.

In the next step (BAT14 analysis) all exhaustion items had item fit residuals within the predefined range and showed no DIF regarding age, gender or country. Residual correlations above the value of 0.13 were indicative of local dependence and were found for items EX1-EX4 0.20, EX3-EX5 0.18 and EX3-EX4 0.15. In the previous step, the decision was made to keep both items EX1 and EX4; so possible candidates for elimination at this stage were items EX3 and EX5. The fit of these items was not bad according to the item fit statistics, even though the residual correlations were slightly higher than expected. Both items were similar in terms of locations. Thus, the decision on which item to delete in the next step was based on content analysis. Item EX5 (*When I get up in the morning, I lack the energy to start a new day at work*) was chosen for elimination since lacking energy in the morning does not necessarily imply that the cause of energy lack is work-related, whereas the content of EX3 (*After a day at work, I find it hard to recover my energy*) is work-related.

In the last step (BAT13 analysis) item EX3 had an item fit residual of 2.9. *None* of the remaining four EX items showed any signs of DIF. Once again, the highest residual correlation was found for the pair EX1-EX4 0.20. Above the critical value of 0.12 were also item pairs EX1-EX3 0.15 and EX3-EX4 0.17. Based on the item fit residual and the residual correlations with the other items, EX3 was noted as possible candidate for exclusion. Moreover, although no misfit was found regarding the item fit indicators, item EX6 (*I want to be active at work, but somehow, I am unable to manage*) was also considered as a candidate for removal based on item threshold locations, presented in Additional file 4 (BAT13). Item EX6 had a somewhat higher location than items EX1, EX3 and EX4, which had the lowest locations of all 13 items. The decision was to keep EX3 and exclude EX6 in the final step, arriving at twelve items to improve the coverage of items across the latent trait.

#### Mental distance

In the BAT23 analysis, items MD1, MD2 and MD3 showed misfit according to the item fit residual and MD2 had a significant item χ^2^ statistic. DIF for gender was observed for item MD4 (women scored lower than men) and for country for item MD2 (FL lower than NL). High residual correlations were observed for many pairs of items. The decision was taken to delete MD2due to misfit on multiple indicators.

In the next step (BAT19 analysis) misfit according to item fit residuals was found for items MD1 and MD3. DIF for gender was observed for item MD4 (women rated lower than men). The highest residual correlation (0.34) was observed between MD1 (*I struggle to find any enthusiasm for my work*) and MD4 (*I feel indifferent about my job*). MD4 was selected for removal, based on DIF issues and the highest correlations with the other items and the subject matter analysis.

#### Cognitive impairment

In the BAT23 analysis, item CI2 had a high negative item fit residual DIF for country was noted for item CI3 (NL rated consistently lower than FL, given the same burnout level). High residual correlations were found for all item pairs. The highest correlation was found between items CI2 (At work I struggle to think clearly) and CI4 (When I’m working, I have trouble concentrating). Although the item CI4 was also a possible candidate according to the item content analysis, the decision was made to remove the item CI2 in the next step due to misfit to multiple item fit indicators.

All CI items have good fit according to the item fit statistics in the second step (BAT19 analysis). DIF between countries was found for item CI3 only (NL rated lower than FL). Residual correlations between all pairs were higher than expected under the condition of local independency. The highest correlation (0.43) was found between items CI1 (*At work, I have trouble staying focused*) and CI4 (*When I’m working, I have trouble concentrating*), and 0.34 between items CI3 (*I am forgetful and distracted at work*) and CI5 (*I make mistakes in my work because I have my mind on other things*), respectively. The decision here was to delete CI3 in the next step because the DIF and the correlation with CI5.

#### Emotional impairment

In the initial analysis (BAT23) the emotional impairment items EI3, EI4 and EI5 had high item fit residuals. Item EI4 also had problems with class intervals and DIF for country. High residual correlations were found between all pairs of EI items, except for EI2-EI3, which was just below the cut-off of > 0.16. The two highest correlations were observed for the item pairs EI2-EI4 and EI1-EI4 respectively. Based on these findings EI4 was removed.

In the next step (BAT19 analysis) items EI3 and EI5 had high item fit residuals. No DIF issues were observed. The residual correlations between all item pairs were above 0.15, except for the pair EI2-EI3, which was 0.15. The highest residual correlation was 0.41 between EI1 (*At work, I feel unable to control my emotions*) and EI2 (*I do not recognize myself in the way I react emotionally at work*) followed by 0.39 between EI-EI5 and 0.33 between EI3-EI5. Item EI5 was also suggested as a candidate for deletion according to the subject matter analysis. It was not an easy decision, so both EI3 and EI5 were tested as candidates to discard in the next step, one at a time. Therefore, in the next step, two combinations of BAT15 items were tested (BAT15a – EI3 deleted and BAT15b – EI5 deleted).

Item fit residuals from both analyses are shown in Table 2. In the BAT15a analysis item EI5 had a high item fit residual and in the BAT15b analysis it was item EI3, which showed a significant χ2 statistic. No DIF issues were noted and residual correlations from the two analyses were comparable in pattern and magnitude (see Additional file 2 and Additional file 3). The summary fit statistics from the two analyses were also comparable, except for the χ2 statistic, which was lower for BAT15a (Table 1). Thus, based on the significant item fit residual for EI3 and the lower χ2 statistic in the BAT15a, the decision was to exclude EI3 and to keep EI5 in the emotional impairment subscale.

**Fig. 1:**
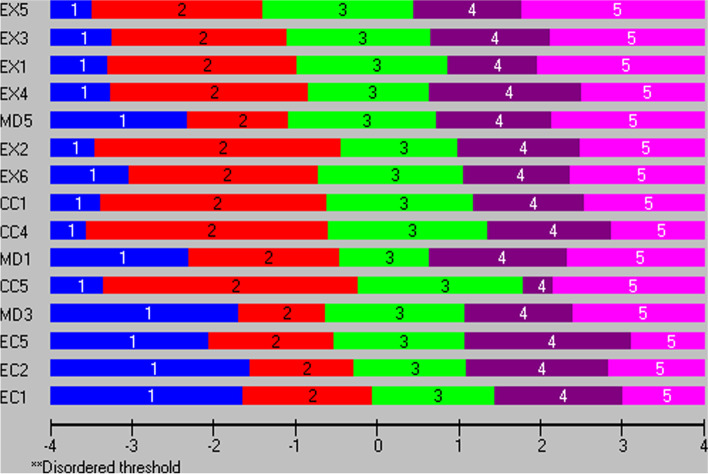
Item hierarchy of the BAT15a items and thresholds ordering on a logit scale (higher value indicates higher burnout) of the Burnout Assessment Tool

**Fig. 2:**
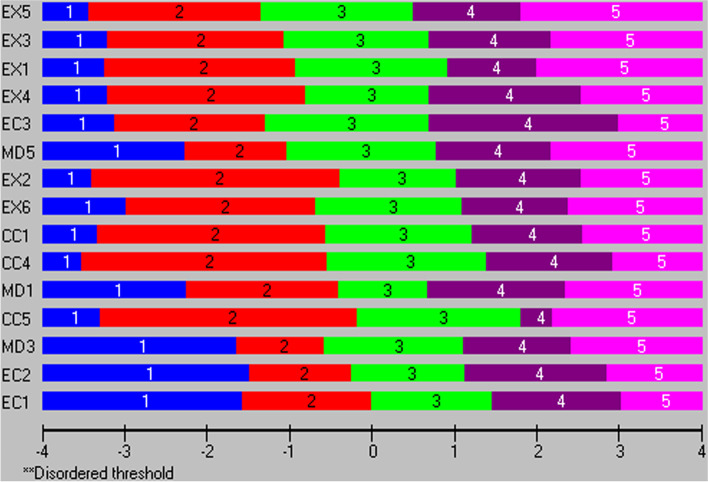
Item hierarchy of the BAT15b items and thresholds ordering on a logit scale (higher value indicates higher burnout) of the Burnout Assessment Tool

Before taking a final decision, item and threshold locations (positionings) on a latent trait were also examined. In Additional file 4 item and threshold locations from both analyses are presented. The same information is visualized in Fig. 1, were items from the BAT15a are rank-ordered from bottom to top, indicating increasing severity according to their locations on the latent burnout continuum (shown at the bottom of the plot ranging from -4 to 4 on a logit scale, with higher values indicating higher levels of burnout). All three EI items are found at the bottom of the list, meaning that they had the highest locations, while the EX items are found at the top. Looking at item thresholds, the intended increasing levels of severity across the response categories (1 = never to 5 = always) is reflected in the data for *all* items; hence the model expected Guttman structure is confirmed. The thresholds’ locations for the EI items are higher than, for example, the EX items, meaning that the highest response categories for the EI items are endorsed at higher levels of the latent burnout trait, compared to the corresponding categories for the EX items. Corresponding results from the BAT15b analysis are shown in Additional file 3 (BAT15b) and Fig. 2. The results displayed in Fig. 2 show that in terms of locations, EI3 is more similar to the EX items than the other two EI items. Therefore, the decision was to keep EI5 instead of EI3, in order to improve the spread of item locations across the latent trait.

### The construct validity of the BAT12

The final step was to test the fit of the BAT12 to the Rasch model. The new BAT12 included three items from each subscale, namely: EX1, EX3, EX4, MD1, MD3, MD5, CI1, CI4, CI5 and EI1, EI2, EI5 (see Additional File 1 for item formulations).

Compared to the initial BAT23 analysis, the item and person fit residuals were at approximately the same levels and were higher than the expected zero and one values, respectively (Table 1). The value of the χ2 statistic has decreased to approximately 160, with a p-value that was still significant. Item MD3 showed DIF for gender but was not further investigated due to problems with local dependency. Problems with local dependency were indicated by the test for unidimensionality, where the lower confidence bound was > 5% and further confirmed by the item residual correlations. As expected, due to the reduction in items and problems with local dependency, the PSI value decreased from 0.95 to 0.91. High item fit residuals were found for items EX3, MD1, MD3 and EI5 (Table 2).

**Fig. 3:**
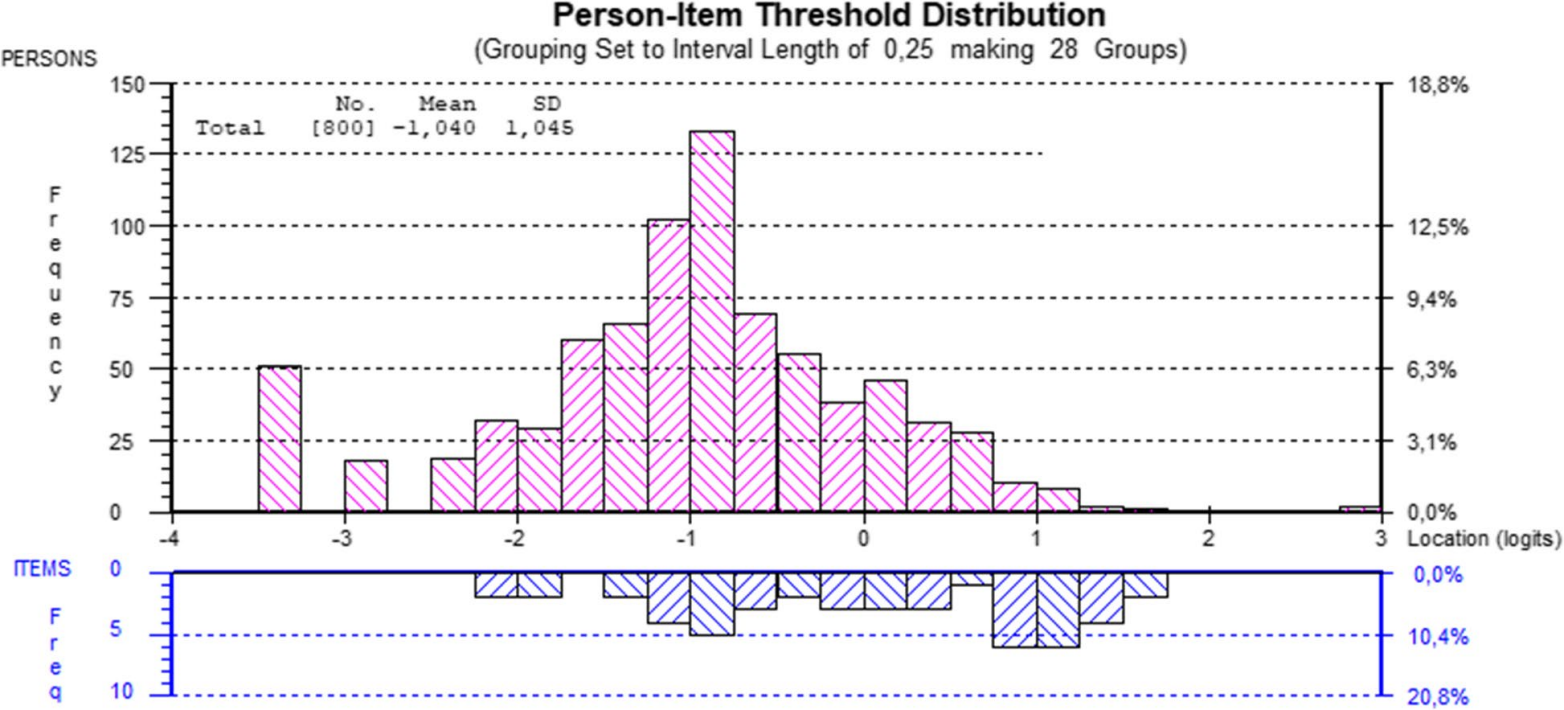
Person and item threshold distribution along the logit scale (higher value indicate higher burnout) within the BAT12 analysis on four testlets of the Burnout Assessment Tool

The pattern of residual correlations mapped to the underlying conceptual structure of the BAT and its four subscales: exhaustion, mental distance, cognitive and emotional impairment (see correlation table in Additional file 2, BAT12). Consequently, to account for the local dependency, the items were grouped into subscale-based testlets, and the analysis was re-run (Table 1, BAT12 4 testlets). The testlets analysis resulted in a good fit to the Rasch model according to the overall fit statistics. Although the number of items was almost halved, the targeting of the BAT12 (Fig. 3) and BAT23 [12] were similar. As seen in Fig. 3, showing the person-item distribution, there is a group of participants with very low burnout levels (-2 on logit scale) and these are lower levels of burnout than measured by the items. The same information is also illustrated by the person mean -1.04 (SD 1.045) compared to the item mean, which is constrained to 0. Thus, the targeting was not optimal, but still acceptable.

The average latent correlation between the four testlets was 0.71, explained common variance was 93%, which is further evidence that the responses on the four subscales can be summarized into a single score.

DIF for gender was found for testlets EX and MD and DIF for country for testlet CC. In the next step, the variable with the highest F-value e.g. MD testlet was split for gender which resulted in a disappearance of gender DIF for the exhaustion testlet and indicated artificial DIF. Artificial DIF is further confirmed by the non-significant difference between the MD location values for women and men in the DIF resolved analysis (0.03 and -0.07 respectively, p-value 0.03). The same procedure was repeated for country DIF and cognitive impairment, also showing artificial DIF (results not shown). The conclusion was that no adjustments for DIF were needed.

## Results, sample 2

The process of shortening from BAT23 to BAT12 was repeated using sample 2, by way of cross-validation. The shortening of the scale resulted in the selection of the same BAT12 items (EX1, EX4, EX5, MD1, MD3, MD5, CI1, CI4, CI5 and EI1, EI2, EI5). In each step, items were chosen for elimination based on the same the criteria and logical reasoning as for sample 1, and therefore not explained in detail here. (Details about this analysis can be obtained upon request from the first author.)

The summary fit statistics and item fit residuals for each step are shown in Tables 3 and 4, respectively. The residual correlations and DIF tables are shown in Additional file 2 and Additional file 3 respectively. As in the sample 1 analyses, the residual correlations mapped into the BATs four subscales and high residual correlations between item pairs belonging to different subscales were *not* observed in any of the analyses. All items had ordered response categories in all analyses.

**Table 3:** Summary fit statistics, subsample 2 (*n* = 800)

	**Item residual**		**Person residual**		**Chi square**		**Unidemensionality**	
**Analysis name**	**Mean**	**SD**	**Mean**	**SD**	**Value**	**p**	**PSI**	**Test % (95% CI)**
BAT19	-0.02	2.05	-0.74	2.25	240.74	< 0.0001	0.94	18.6 (16.0;21.5)
BAT15	0.02	1.94	-0.71	2.07	190.76	0.001	0.93	14.5 (12.2;17.2)
BAT14	0.04	1.64	-0.69	1.98	163.02	0.02	0.92	14.9 (12.6;17.7)
BAT13	-0.02	1.66	-0.66	1.88	183.13	< 0.0001	0.91	14.6 (12.2;17.3)
BAT12	-0.07	1.96	-0.67	1.85	163.31	0.0004	0.90	14.1 (11.8;16.8)
BAT12 4 testlets	0.31	0.80	-0.52	1.13	57.67	0.01	0.81	4.3 (3.0;5.8)

**Table 4:** Item fit residuals, subsample 2 (*n* = 800). Bold indicates significant item chi square (Bonferroni adjusted)

**Item**	**BAT19**	**BAT15**	**BAT14**	**BAT13**	**BAT12**
EX1	-0.69	-0.60	-0.59	-0.52	-0.06
EX2	-1.35	-1.42	-1.40		
EX3	1.17	1.39	1.32	1.49	2.27
EX4	0.35	0.40	0.35	0.97	1.47
EX5	0.24	0.20	0.47	0.64	
EX6	-3.66	-3.71			
EX7	2.78				
EX8					
MD1	-0.21	0.58	0.38	0.09	0.23
MD2					
MD3	-1.54	-1.24	-1.74	-1.87	-1.68
MD4	2.56				
MD5	3.86	4.35	3.64	3.10	2.98
CI1	-0.80	-0.26	-0.47	-0.67	-0.70
CI2					
CI3	2.00				
CI4	-0.76	-0.22	-0.23	-0.53	-0.50
CI5	1.16	1,88	1.51	1.19	1.00
EI1	-2.28	-1.59	-2.06	-2.48	-3.18
EI2	-2.47	-1.71	-2.21	-2.65	-3.30
EI3					
EI4	-2.44				
EI5	1.76	2.19	1.58	0.96	0.67

The average latent correlation between the four testlets (0.67) explained 92% of the variance of the BAT12.

## Ordinal-to-interval conversion table

Person scores from the Rasch analysis are used to transform the mean values of the BAT12 which are ordinal scores, into metric, interval-level scores. This was possible given the good fit of the BAT12 to the Rasch model after accounting for local dependency. Person scores can take both negative and positive values since they are situated on a logit scale, which can be hard to interpret compared to the original 1–5 range. For that reason, the logit person scores are linearly transformed into 1–5 interval scores. In Table 5 we provided interval scores in both logit units and in a 1–5 range, allowing users of the BAT to convert the ordinal mean score into interval-level (metric) scores. The total sample (*n* = 2,978) was used for the score calculation which increases the precision of the scores.

**Table 5:** Conversion table with raw mean scores (ordinal) on the BAT12, short version of the Burnout Assessment Tool and their corresponding interval scale metric and logit equivalents based on Rasch analysis (*n* = 2,978)

**Mean**	**Metric**	**Logit**		**Mean**	**Metric**	**Logit**
1.00	1.00	-3.40		3.28	3.08	0.17
1.41	1.08	-2.76		3.33	3.17	0.25
1.68	1.17	-2.34		3.39	3.25	0.33
1.86	1.25	-2.06		3.44	3.33	0.41
1.99	1.33	-1.85		3.49	3.42	0.49
2.10	1.42	-1.68		3.54	3.50	0.57
2.19	1.50	-1.53		3.58	3.58	0.64
2.27	1.58	-1.41		3.63	3.67	0.71
2.35	1.67	-1.29		3.67	3.75	0.78
2.41	1.75	-1.19		3.72	3.83	0.85
2.47	1.83	-1.10		3.76	3.92	0.92
2.53	1.92	-1.01		3.81	4.00	0.99
2.58	2.00	-0.92		3.85	4.08	1.06
2.64	2.08	-0.84		3.90	4.17	1.13
2.69	2.17	-0.76		3.95	4.25	1.21
2.74	2.25	-0.68		4.00	4.33	1.29
2.79	2.33	-0.60		4.05	4.42	1.37
2.84	2.42	-0.52		4.11	4.50	1.47
2.90	2.50	-0.44		4.18	4.58	1.57
2.95	2.58	-0.35		4.25	4.67	1.69
3.00	2.67	-0.27		4.35	4.75	1.84
3.06	2.75	-0.18		4.48	4.83	2.04
3.11	2.83	-0.10		4.68	4.92	2.35
3.17	2.92	-0.01		5.00	5.00	2.85
3.22	3.00	0.08				

## Discussion

The aim of this study was fourfold. Combining quantitative (Rasch analysis) and qualitative approaches (subject matter analysis and expert judgement), the original 23-item version of the BAT (BAT23) was shortened to 12 items (BAT12), which was the first aim. Using the Rasch analysis, the BAT12 construct validity consisting of the four subscales was evaluated in the second aim. The results showed that the BAT12 has good psychometric properties after adjusting for local dependency between the items within each subscale. The BAT12 fulfils the criteria required by the Rasch measurement model when subscales are used instead of item scores. Thus, the BAT12 quantifies a latent trait of burnout. The results show that the items of the BAT12, like those of the BAT23, reflect the scoring structure indicated by the developers of the scale and the BAT’s four subscales can be summarized into a single burnout score (third aim). The BAT12 works in the same way (invariantly) for women and men, younger and older and across both countries, meaning that the fourth aim was also achieved. Finally, given good fit to the Rasch model, the mean scores of the BAT12 have been transformed into interval metric scores, which allows the use of parametric statistical techniques.

## Shortening of the scale

Shortening of questionnaires in a proper way and testing different aspects of validity is an important, yet often neglected issue [23]. For time saving reasons, survey researchers are often forced to develop the shorter scales themselves, which gives rise to several problems. First, psychometric properties of these newly developed instruments are not always tested or available for a larger audience. Secondly, several short versions might be developed, which hinders comparisons between different populations and over time. Thirdly, in some cases the psychometric properties of the original version of the instrument could also be questioned.

The advantage of the BAT12 established in the present study, is that the original version of the BAT was developed as an alternative self-report questionnaire that effectively addressed the conceptual and technical difficulties associated with the MBI, the most commonly used instrument to measure burnout. Incorporating the results of the intensive burnout literature during the past four decades, along with clinical experiences, an updated burnout definition was presented [10]. This resulted in a new measure: the Burnout Assessment Tool (BAT). The unique feature of this newly developed measure is that the BAT has a strong conceptual basis, promising psychometric properties and that the instrument has been anchored to be used globally [11].

In a literature review regarding scale-shortening strategies, Kruyen et al. highlighted that researchers often aim to maximize the reliability coefficient [21]. Cronbach’s alpha is probably the most reported reliability coefficient of internal consistency, and the shortening strategies are often aiming to achieve a coefficient alpha above a certain value as prescribed by rules of thumbs. This is of course not unproblematic. Strategies involving maximizing the alpha coefficient result in selecting items that are highly correlated, excluding lowly correlating Items. Highly correlated items are however often similar in content. This way of selecting items tends to narrow construct coverage. Moreover, items that are similar in content might cause a bias, when respondents noticing these similarities purposively (but incorrectly) match the responses from similar items to ensure response consistency [21]. In contrast, the indication of local dependency among items, i.e., high residual correlation between items, was one of the criteria for the elimination of items in this study. The strength of this study is that the focus was not on optimizing the reliability coefficient, but to ensure broad construct coverage. Moreover, the number of items (three in each subscale) was predefined and not decided based on the value of the reliability coefficient.

## Psychometric properties of the BAT12

As mentioned, the goal of the Rasch analysis is to evaluate the fit of the data to the Rasch model, which implies testing of several assumptions. If a scale works properly, estimates of thresholds need to be ordered. Thresholds are partitioning the latent continuum of burnout into ordered categories and the increasing levels of burnout should be consistent with the response ordering of the items. All BAT12 items had ordered thresholds, meaning that the increasing level of burnout severity across the categories was reflected in the data for all BAT12 items. In other words, respondents are using the item response categories (from never to always) as intended by the developers.

Evaluation of local dependency and unidimensionality are crucial steps in the evaluation of scales. Residual correlations between the items revealed that the items clustered within the predefined four subscales in the initial analysis including 12 separate items. Thus, local dependency was present, and the Smith’s test indicated multidimensionality. These results are consistent with previous studies on the BAT23 and with the theoretical conceptualisation of burnout as a syndrome [10–12].

Although the results were expected and made perfect sense theoretically, local dependency was still a problem from a measurement viewpoint [50]. We have accounted for local dependency by combining the items from each subscale into four testlets [42–44]. When items within each subscale were combined into four testlets, fit to the Rasch model was achieved (BAT12 4 testlets analysis). The high explained common variance and average latent correlation between the four testlets indicated a strong general factor, which is a prerequisite for combining the items into a single burnout score. Thus, the BAT12 quantifies a latent trait of burnout in the same way as the BAT23. In other words, burnout is illustrated as a syndrome with four interrelated symptoms that all refer to one underlying mental state. The responses of the four subscales can thus be summarized into a single burnout score.

Finally, evaluation of DIF is an equally important step in a scale validation process. In a frame of reference that includes different groups, it is important to investigate whether the scale works invariantly across these different groups and thus can be used for comparison between the groups. In this study we have instigated DIF for gender, age and country and found that no adjustments for DIF were needed. Thus, as was the case for the BAT23, also the BAT12 works invariantly for women and men, older and younger and for participants from both countries.

## Strengths and limitations

One strength of the present study is that the data come from large, representative samples of the working population in the Netherlands and Flanders (Belgium). On the other hand, when using chi-square statistics in statistical inference, large sample sizes could result in bias, as even minor levels of misfit become statistically significant. We tried to solve this issue by selecting two random samples from the dataset with 800 participants each, which were still large enough to perform the statistical analyses with good precision. In addition, we were able to cross-validate results in both samples. If there had been major problems with the scale, these would have emerged in both subsamples.

The focus on the content coverage of the BAT12 was mentioned as a strength. The combination of different approaches is another strength. Various item indicators and positioning of the items along the burnout continuum were considered as part of the statistical strategy. Through subject matter analysis the items were classified into predefined categories and items that were judged to be similar in content were considered as candidates for deletion. To further ensure the broad construct coverage, the meaning and content of the items were evaluated by experts (i.e., the developers of the BAT) and items that were judged theoretically important were kept in the final selection. The decision on which items to include in a short version of an instrument should not be solely made on psychometric properties, but also needs to incorporate strong theoretical considerations [18].

As mentioned above, previous studies regarding different validity aspects of the BAT23 showed promising results. A limitation is that these validity results do not automatically transfer to the short version of BAT12 but need to be investigated in forthcoming studies. Another limitation is that the participants in this study have answered all 23 items. As recommended in the literature [25], the psychometric results should be further validated in a new study using the BAT12 items only. To the best of our knowledge, so far the psychometric properties of the BAT12 were evaluated in two studies, both with promising results [51, 52], but more studies are needed.

Besides the psychometric properties of the short scale, another crucial issue is the scale’s invariance property under the reduced number of items, i.e., whether the same conclusion about the latent construct (burnout) can be drawn irrespective of whether the short or long version is used. This is especially important for individual assessments as the shortening of a scale affects the accuracy and precision of the scores. In this study the PSI was 0.82 and 0.81 in the sample 1 and sample 2 respectively and 0.82 in the total sample. These values were somewhat lower compared to the BAT23 the four testlets analysis (0.85) [12] but still high enough to allow comparison of the BAT respondents with high precision on both group and individual levels. However, future studies of the BAT12 should further focus on this issue.

## Practical use of the BAT12

The intended use of the (short) scale needs to be specified and discussed, e.g. whether scales are intended to be used for research, screening, clinical assessments etc.[26, 27]. A shorter version of the BAT is timesaving compared to the BAT23 and can be used in e.g. employee surveys for screening purposes. Shorter scales are typically used in larger survey assessment in organizations, as many variables need to be measured in order to comply with the legal requirements to perform a work-related psycho-social risk analysis. Longer scales are less practical in that regard and could be used for a more detailed assessment of individuals, needed for their clinical follow-up. The construction of a scale and its evaluation requires samples that match the intended target population. In this study, data from representative samples of the working population in the Netherlands and Flanders (Belgium) was used. As regards the diagnostic or clinical use of the BAT23, studies so far analyzed and reported clinical cut-off values [29]. The results of the present study indicate good precision when measuring burnout complaints, suggesting that individual assessment of burnout can also be done using the BAT12. However, we recommend to additionally perform studies on patients to assess the classification consistency of the BAT12, and to establish clinical cut-off values.

Given the good fit to the Rasch model and acceptable targeting, an ordinal-to-interval-conversion table is reported in this study. For the users of the BAT12 we recommend the use of metric scores instead of mean scores. In this way better precision can be obtained. The reason is that the mean scores are calculated using ordinal response categories. They are thus not equidistant across the entire continuum that is being measured. This means that the increase of one unit does not imply the same burnout magnitude along the entire burnout continuum. This problem is more pronounced toward both ends of the scale and perhaps not that serious in the middle of the scale. Interestingly, it is toward the upper end of the scale that we would expect to find people at high risk of burnout. This is obviously a well-known issue that is true for many ordinal scales and not in any way unique for the BAT [53].

Regarding the practical implications for the total burnout score, we recommend the users of the BAT12 to first calculate the mean scores for each person and then translate these into metric scores using the conversion table. Metric score variables can be used in further analyses (e.g., calculation of population average levels). The conversion table is valid for complete answers only (no missing values are allowed).

## Conclusion

Using data from representative samples of working populations in the Netherlands and Flanders (Belgium) and combining quantitative and qualitative approaches, a new shorter version of the BAT (Burnout Assessment Tool) – BAT12 -is developed and tested psychometrically. The new BAT12 developed in the present study maintains the breath of item content of the original version of the BAT. The shorter version of the BAT has sound psychometric properties. The BAT12 fulfils the measurement criteria according to the Rasch model when the four subscales are combined into a single burnout score. The scale works invariantly for women and men, older and younger and across both countries. A shorter version of the BAT is timesaving compared to the BAT23 and can be used in e.g., employee surveys.

## Supplementary Information

**Additional file 1.**

**Additional file 2.**

**Additional file 3.**

**Additional file 4.**

**Additional file 5.**

**Additional file 6.**

**Additional file 7.**

**Additional file 8.**

## Data Availability

All data generated or analysed during this study are included in this published article [and its supplementary information files].

## Abbreviations

ANOVA: Analysis of variance
BAT: Burnout Assessment Tool
DIF: Differential item functioning
FL: Flanders
MBI: The Maslach Burnout Inventory
NL: The Netherlands
PCA: Principal component analysis
PSI: Person Separation Index
