# Supplementary material for: Shortening of the Burnout Assessment Tool (BAT)—from 23 to 12 items using content and Rasch analysis
Source: BMC Public Health. 2022 Mar 22;22:560. doi: 10.1186/s12889-022-12946-y (PMC8939057; doi:10.1186/s12889-022-12946-y)
Supplement: Supplementary file 1 — Additional file 1. [file 12889_2022_12946_MOESM1_ESM.pdf]

Work-related version of the BAT

Instruction

The following statements are related to your work situation and how you experience this situation. Please state how often each statement applies to you.

Scoring

Never	Rarely	Sometimes	Often	Always
1	2	3	4	5

Core symptoms

	Never	Rarely	Sometimes	Often	Always
Exhaustion					
1. At work, I feel mentally exhausted*	☐	☐	☐	☐	☐
2. Everything I do at work requires a great deal of effort	☐	☐	☐	☐	☐
3. After a day at work, I find it hard to recover my energy*	☐	☐	☐	☐	☐
4. At work, I feel physically exhausted*	☐	☐	☐	☐	☐
5. When I get up in the morning, I lack the energy to start a new day at work	☐	☐	☐	☐	☐
6. I want to be active at work, but somehow I am unable to manage	☐	☐	☐	☐	☐
7. When I exert myself at work, I quickly get tired	☐	☐	☐	☐	☐
8. At the end of my working day, I feel mentally exhausted and drained	☐	☐	☐	☐	☐
Mental distance					
9. I struggle to find any enthusiasm for my work*	☐	☐	☐	☐	☐
10. At work, I do not think much about what I am doing and I function on autopilot	☐	☐	☐	☐	☐
11. I feel a strong aversion towards my job*	☐	☐	☐	☐	☐
12. I feel indifferent about my job	☐	☐	☐	☐	☐
13. I'm cynical about what my work means to others*	☐	☐	☐	☐	☐

Citation: Schaufeli, W.B., De Witte, H. & Desart, S. (2019). *Burnout Assessment Tool (BAT) – Test Manual.* KU Leuven, Belgium: Internal report.

	Never	Rarely	Sometimes	Often	Always
Cognitive impairment					
14. At work, I have trouble staying focused*	☐	☐	☐	☐	☐
15. At work I struggle to think clearly	☐	☐	☐	☐	☐
16. I'm forgetful and distracted at work	☐	☐	☐	☐	☐
17. When I'm working, I have trouble concentrating*	☐	☐	☐	☐	☐
18. I make mistakes in my work because I have my mind on other things*	☐	☐	☐	☐	☐
Emotional impairment					
19. At work, I feel unable to control my emotions*	☐	☐	☐	☐	☐
20. I do not recognize myself in the way I react emotionally at work*	☐	☐	☐	☐	☐
21. During my work I become irritable when things don't go my way	☐	☐	☐	☐	☐
22. I get upset or sad at work without knowing why	☐	☐	☐	☐	☐
23. At work I may overreact unintentionally*	☐	☐	☐	☐	☐

Note: * = Short version

Citation: Schaufeli, W.B., De Witte, H. & Desart, S. (2019). *Burnout Assessment Tool (BAT) – Test Manual.* KU Leuven, Belgium: Internal report.
