# Supplementary material for: Shortening of the Burnout Assessment Tool (BAT)—from 23 to 12 items using content and Rasch analysis
Source: BMC Public Health. 2022 Mar 22;22:560. doi: 10.1186/s12889-022-12946-y (PMC8939057; doi:10.1186/s12889-022-12946-y)
Supplement: Supplementary file 2 — Additional file 2. [file 12889_2022_12946_MOESM2_ESM.pdf]

# Appendix 2 Observed correlation matrixes for the Burnout Assessment Tool.

**Table S1. Analysis name BAT19 containing 19 items, representative sample of working population NL and FL subsample 1 n=800.**

| Item | EX1 | EX2 | EX3 | EX4 | EX5 | EX6 | EX7 | MD1 | MD3 | MD4 | MD5 | CI1 | CI3 | CI4 | CI5 | EI1 | EI2 | EI3 | EI5 |
|---|---|---|---|---|---|---|---|---|---|---|---|---|---|---|---|---|---|---|---|
| **EX1** | 1.00 | | | | | | | | | | | | | | | | | | |
| **EX2** | .06 | 1.00 | | | | | | | | | | | | | | | | | |
| **EX3** | .16 | .06 | 1.00 | | | | | | | | | | | | | | | | |
| **EX4** | .23 | .07 | .17 | 1.00 | | | | | | | | | | | | | | | |
| **EX5** | .02 | .11 | .20 | .08 | 1.00 | | | | | | | | | | | | | | |
| **EX6** | .03 | .02 | .03 | .09 | .05 | 1.00 | | | | | | | | | | | | | |
| **EX7** | .08 | .03 | .11 | .18 | -.02 | .03 | 1.00 | | | | | | | | | | | | |
| **MD1** | -.08 | -.03 | -.16 | -.11 | -.03 | .02 | -.23 | 1.00 | | | | | | | | | | | |
| **MD3** | .01 | .04 | -.10 | -.07 | -.03 | -.04 | -.15 | .28 | 1.00 | | | | | | | | | | |
| **MD4** | -.18 | -.01 | -.23 | -.18 | -.09 | -.07 | -.19 | .34 | .30 | 1.00 | | | | | | | | | |
| **MD5** | -.09 | -.06 | -.07 | -.13 | -.05 | -.09 | -.16 | .22 | .14 | .26 | 1.00 | | | | | | | | |
| **CI1** | -.12 | -.11 | -.22 | -.18 | -.13 | -.10 | -.06 | -.05 | -.12 | -.09 | -.15 | 1.00 | | | | | | | |
| **CI3** | -.17 | -.12 | -.21 | -.15 | -.16 | -.04 | -.10 | -.14 | -.21 | -.10 | -.15 | .31 | 1.00 | | | | | | |
| **CI4** | -.13 | -.10 | -.25 | -.15 | -.11 | -.05 | -.12 | -.13 | -.18 | -.10 | -.13 | .43 | .33 | 1.00 | | | | | |
| **CI5** | -.21 | -.18 | -.20 | -.23 | -.18 | -.10 | -.01 | -.15 | -.17 | -.09 | -.20 | .22 | .34 | .28 | 1.00 | | | | |
| **EI1** | -.14 | -.12 | -.10 | -.20 | -.18 | -.16 | -.14 | -.17 | -.13 | -.16 | -.12 | -.07 | -.11 | -.06 | -.02 | 1.00 | | | |
| **EI2** | -.14 | -.15 | -.16 | -.16 | -.28 | -.15 | -.16 | -.16 | -.08 | -.07 | -.13 | -.09 | -.04 | -.08 | .05 | .41 | 1.00 | | |
| **EI3** | -.14 | -.17 | -.09 | -.17 | -.14 | -.21 | -.16 | -.16 | -.20 | -.12 | -.06 | -.11 | -.05 | -.08 | .03 | .19 | .15 | 1.00 | |
| **EI5** | -.18 | -.20 | -.14 | -.19 | -.22 | -.17 | -.15 | -.16 | -.16 | -.15 | -.12 | -.12 | -.07 | -.11 | .00 | .39 | .34 | .33 | 1.00 |

**Table S2. Analysis name BAT15a containing 15 items, representative sample of working population NL and FL subsample 1 n=800.**

| Item | EX1 | EX2 | EX3 | EX4 | EX5 | EX6 | MD1 | MD3 | MD5 | CI1 | CI4 | CI5 | EI1 | EI2 | EI5 |
|---|---|---|---|---|---|---|---|---|---|---|---|---|---|---|---|
| **EX1** | 1.00 | | | | | | | | | | | | | | |
| **EX2** | .03 | 1.00 | | | | | | | | | | | | | |
| **EX3** | .13 | .03 | 1.00 | | | | | | | | | | | | |
| **EX4** | .19 | .04 | .14 | 1.00 | | | | | | | | | | | |
| **EX5** | -.02 | .08 | .17 | .04 | 1.00 | | | | | | | | | | |
| **EX6** | -.01 | -.01 | -.01 | .06 | .02 | 1.00 | | | | | | | | | |
| **MD1** | -.11 | -.06 | -.20 | -.14 | -.05 | .00 | 1.00 | | | | | | | | |
| **MD3** | -.03 | .02 | -.13 | -.10 | -.07 | -.07 | .28 | 1.00 | | | | | | | |
| **MD5** | -.12 | -.07 | -.09 | -.15 | -.07 | -.10 | .21 | .12 | 1.00 | | | | | | |
| **CI1** | -.14 | -.12 | -.24 | -.19 | -.15 | -.11 | -.05 | -.13 | -.14 | 1.00 | | | | | |
| **CI4** | -.15 | -.11 | -.27 | -.16 | -.13 | -.06 | -.12 | -.19 | -.13 | .44 | 1.00 | | | | |
| **CI5** | -.21 | -.17 | -.20 | -.22 | -.18 | -.09 | -.13 | -.16 | -.17 | .24 | .29 | 1.00 | | | |
| **EI1** | -.17 | -.15 | -.14 | -.22 | -.22 | -.18 | -.19 | -.15 | -.14 | -.07 | -.07 | -.01 | 1.00 | | |
| **EI2** | -.17 | -.17 | -.19 | -.17 | -.31 | -.17 | -.18 | -.09 | -.14 | -.09 | -.08 | .07 | .39 | 1.00 | |
| **EI5** | -.19 | -.21 | -.16 | -.20 | -.24 | -.17 | -.17 | -.17 | -.11 | -.11 | -.10 | .02 | .39 | .34 | 1.00 |

**Table S3. Analysis name BAT15b containing 15 items, representative sample of working population NL and FL subsample 1 n=800.**

| Item | EX1 | EX2 | EX4 | EX4 | EX5 | EX6 | MD1 | MD3 | MD5 | CI1 | CI4 | CI5 | EI1 | EI2 | EI3 |
|---|---|---|---|---|---|---|---|---|---|---|---|---|---|---|---|
| **EX1** | 1.00 | | | | | | | | | | | | | | |
| **EX2** | .02 | 1.00 | | | | | | | | | | | | | |
| **EX3** | .12 | .02 | 1.00 | | | | | | | | | | | | |
| **EX4** | .19 | .04 | .14 | 1.00 | | | | | | | | | | | |
| **EX5** | -.03 | .08 | .16 | .03 | 1.00 | | | | | | | | | | |
| **EX6** | -.01 | -.01 | -.01 | .06 | .01 | 1.00 | | | | | | | | | |
| **MD1** | -.12 | -.06 | -.20 | -.14 | -.06 | .00 | 1.00 | | | | | | | | |
| **MD3** | -.03 | .02 | -.14 | -.10 | -.07 | -.06 | .28 | 1.00 | | | | | | | |
| **MD5** | -.13 | -.08 | -.10 | -.16 | -.08 | -.11 | .20 | .11 | 1.00 | | | | | | |
| **CI1** | -.14 | -.12 | -.24 | -.19 | -.16 | -.11 | -.05 | -.13 | -.15 | 1.00 | | | | | |
| **CI4** | -.15 | -.12 | -.28 | -.17 | -.13 | -.06 | -.13 | -.19 | -.14 | .43 | 1.00 | | | | |
| **CI5** | -.22 | -.17 | -.21 | -.22 | -.18 | -.09 | -.14 | -.16 | -.18 | .24 | .29 | 1.00 | | | |
| **EI1** | -.16 | -.13 | -.12 | -.21 | -.21 | -.16 | -.17 | -.13 | -.13 | -.05 | -.05 | .01 | 1.00 | | |
| **EI2** | -.16 | -.15 | -.18 | -.16 | -.31 | -.15 | -.16 | -.07 | -.13 | -.07 | -.07 | .08 | .41 | 1.00 | |
| **EI3** | -.17 | -.19 | -.12 | -.18 | -.17 | -.21 | -.16 | -.20 | -.05 | -.10 | -.08 | .05 | .20 | .17 | 1.00 |

**Table S4. Analysis name BAT14 containing 14 items, representative sample of working population NL and FL subsample 1 n=800.**

| Item | EX1 | EX3 | EX4 | EX5 | EX6 | MD1 | MD3 | MD5 | CI1 | CI4 | CI5 | EI1 | EI2 | EI5 |
|---|---|---|---|---|---|---|---|---|---|---|---|---|---|---|
| EX1 | 1.00 | | | | | | | | | | | | | |
| EX3 | .13 | 1.00 | | | | | | | | | | | | |
| EX4 | .20 | .15 | 1.00 | | | | | | | | | | | |
| EX5 | -.01 | .18 | .05 | 1.00 | | | | | | | | | | |
| EX6 | .00 | .00 | .07 | .02 | 1.00 | | | | | | | | | |
| MD1 | -.11 | -.20 | -.14 | -.05 | .00 | 1.00 | | | | | | | | |
| MD3 | -.02 | -.13 | -.10 | -.06 | -.06 | .28 | 1.00 | | | | | | | |
| MD5 | -.12 | -.09 | -.14 | -.07 | -.10 | .21 | .11 | 1.00 | | | | | | |
| CI1 | -.14 | -.25 | -.19 | -.15 | -.11 | -.06 | -.13 | -.15 | 1.00 | | | | | |
| CI4 | -.15 | -.27 | -.16 | -.13 | -.06 | -.13 | -.19 | -.14 | .43 | 1.00 | | | | |
| CI5 | -.22 | -.21 | -.22 | -.18 | -.10 | -.14 | -.17 | -.19 | .23 | .28 | 1.00 | | | |
| EI1 | -.17 | -.14 | -.23 | -.22 | -.19 | -.20 | -.16 | -.16 | -.08 | -.08 | -.02 | 1.00 | | |
| EI2 | -.18 | -.20 | -.17 | -.32 | -.17 | -.19 | -.09 | -.15 | -.10 | -.10 | .06 | .38 | 1.00 | |
| EI5 | -.20 | -.16 | -.21 | -.24 | -.19 | -.18 | -.18 | -.13 | -.13 | -.12 | .00 | .38 | .32 | 1.00 |

**Table S5. Analysis name BAT13 containing 13 items, representative sample of working population NL and FL subsample 1 n=800.**

| Item | EX1 | EX3 | EX4 | EX6 | MD1 | MD3 | MD5 | CI1 | CI4 | CI5 | EI1 | EI2 | EI5 |
|---|---|---|---|---|---|---|---|---|---|---|---|---|---|
| **EX1** | 1.00 | | | | | | | | | | | | |
| **EX3** | .15 | 1.00 | | | | | | | | | | | |
| **EX4** | .20 | .17 | 1.00 | | | | | | | | | | |
| **EX6** | .01 | .02 | .08 | 1.00 | | | | | | | | | |
| **MD1** | -.11 | -.18 | -.13 | .00 | 1.00 | | | | | | | | |
| **MD3** | -.02 | -.10 | -.09 | -.06 | .28 | 1.00 | | | | | | | |
| **MD5** | -.12 | -.08 | -.14 | -.10 | .21 | .11 | 1.00 | | | | | | |
| **CI1** | -.15 | -.24 | -.19 | -.12 | -.07 | -.14 | -.17 | 1.00 | | | | | |
| **CI4** | -.15 | -.26 | -.16 | -.07 | -.14 | -.19 | -.16 | .42 | 1.00 | | | | |
| **CI5** | -.23 | -.20 | -.23 | -.11 | -.16 | -.18 | -.20 | .21 | .27 | 1.00 | | | |
| **EI1** | -.19 | -.14 | -.24 | -.20 | -.22 | -.17 | -.17 | -.11 | -.11 | -.05 | 1.00 | | |
| **EI2** | -.21 | -.20 | -.19 | -.20 | -.21 | -.12 | -.18 | -.14 | -.13 | .03 | .35 | 1.00 | |
| **EI5** | -.22 | -.17 | -.22 | -.20 | -.20 | -.20 | -.15 | -.16 | -.15 | -.02 | .37 | .30 | 1.00 |

**Table S6. Analysis name BAT12 containing 12 items, representative sample of working population NL and FL subsample 1 n=800.**

| Item | EX1 | EX3 | EX4 | MD1 | MD3 | MD5 | CI1 | CI4 | CI5 | EI1 | EI2 | EI5 |
|---|---|---|---|---|---|---|---|---|---|---|---|---|
| **EX1** | 1.00 | | | | | | | | | | | |
| **EX3** | .16 | 1.00 | | | | | | | | | | |
| **EX4** | .21 | .19 | 1.00 | | | | | | | | | |
| **MD1** | -.10 | -.17 | -.12 | 1.00 | | | | | | | | |
| **MD3** | -.02 | -.10 | -.08 | .28 | 1.00 | | | | | | | |
| **MD5** | -.12 | -.08 | -.14 | .21 | .11 | 1.00 | | | | | | |
| **CI1** | -.15 | -.24 | -.19 | -.07 | -.16 | -.18 | 1.00 | | | | | |
| **CI4** | -.15 | -.26 | -.16 | -.14 | -.20 | -.16 | .42 | 1.00 | | | | |
| **CI5** | -.23 | -.21 | -.23 | -.16 | -.19 | -.22 | .20 | .27 | 1.00 | | | |
| **EI1** | -.20 | -.15 | -.25 | -.23 | -.19 | -.20 | -.13 | -.13 | -.06 | 1.00 | | |
| **EI2** | -.22 | -.21 | -.20 | -.22 | -.14 | -.20 | -.16 | -.14 | .01 | .34 | 1.00 | |
| **EI5** | -.24 | -.18 | -.23 | -.21 | -.22 | -.18 | -.18 | -.16 | -.04 | .36 | .28 | 1.00 |

**Table S7. Analysis name BAT19 containing 19 items, representative sample of working population NL and FL subsample 2 n=800.**

| Item | EX1 | EX2 | EX3 | EX4 | EX5 | EX6 | EX7 | MD1 | MD3 | MD4 | MD5 | CI1 | CI3 | CI4 | CI5 | EI1 | EI2 | EI4 | EI5 |
|---|---|---|---|---|---|---|---|---|---|---|---|---|---|---|---|---|---|---|---|
| **EX1** | 1.00 | | | | | | | | | | | | | | | | | | |
| **EX2** | .03 | 1.00 | | | | | | | | | | | | | | | | | |
| **EX3** | .11 | .08 | 1.00 | | | | | | | | | | | | | | | | |
| **EX4** | .15 | .18 | .30 | 1.00 | | | | | | | | | | | | | | | |
| **EX5** | .03 | .05 | .18 | .08 | 1.00 | | | | | | | | | | | | | | |
| **EX6** | .08 | .09 | .05 | .05 | .18 | 1.00 | | | | | | | | | | | | | |
| **EX7** | .14 | .11 | .22 | .28 | .01 | -.01 | 1.00 | | | | | | | | | | | | |
| **MD1** | -.12 | -.06 | -.22 | -.15 | -.05 | -.04 | -.25 | 1.00 | | | | | | | | | | | |
| **MD3** | -.04 | .01 | -.17 | -.10 | .00 | -.09 | -.23 | .47 | 1.00 | | | | | | | | | | |
| **MD4** | -.18 | -.07 | -.21 | -.21 | -.05 | -.06 | -.24 | .49 | .41 | 1.00 | | | | | | | | | |
| **MD5** | -.13 | -.12 | -.19 | -.20 | -.10 | -.15 | -.16 | .35 | .28 | .34 | 1.00 | | | | | | | | |
| **CI1** | -.12 | -.09 | -.12 | -.21 | -.10 | -.06 | -.11 | -.18 | -.20 | -.18 | -.16 | 1.00 | | | | | | | |
| **CI3** | -.15 | -.23 | -.15 | -.19 | -.13 | -.12 | -.15 | -.20 | -.27 | -.19 | -.16 | .28 | 1.00 | | | | | | |
| **CI4** | -.16 | -.16 | -.12 | -.24 | -.08 | .01 | -.14 | -.18 | -.24 | -.19 | -.18 | .48 | .34 | 1.00 | | | | | |
| **CI5** | -.14 | -.17 | -.17 | -.21 | -.17 | -.14 | -.08 | -.26 | -.26 | -.23 | -.17 | .23 | .40 | .27 | 1.00 | | | | |
| **EI1** | -.14 | -.17 | -.18 | -.17 | -.30 | -.16 | -.15 | -.21 | -.18 | -.20 | -.13 | -.06 | .04 | -.01 | .09 | 1.00 | | | |
| **EI2** | -.11 | -.14 | -.21 | -.16 | -.26 | -.15 | -.14 | -.19 | -.17 | -.17 | -.13 | -.06 | -.01 | -.03 | .07 | .43 | 1.00 | | |
| **EI4** | -.19 | -.14 | -.16 | -.22 | -.20 | -.20 | -.16 | -.12 | -.10 | -.11 | -.12 | -.07 | -.03 | -.10 | .00 | .43 | .41 | 1.00 | |
| **EI5** | -.11 | -.20 | -.18 | -.13 | -.20 | -.17 | -.15 | -.21 | -.23 | -.18 | -.12 | -.10 | .04 | -.06 | .08 | .42 | .38 | .40 | 1.00 |

**Table S8. Analysis name BAT15 containing 15 items, representative sample of working population NL and FL subsample 2 n=800.**

| **Item** | **EX1** | **EX2** | **EX3** | **EX4** | **EX5** | **EX6** | **MD1** | **MD3** | **MD5** | **CI1** | **CI4** | **CI5** | **EI1** | **EI2** | **EI5** |
|---|---|---|---|---|---|---|---|---|---|---|---|---|---|---|---|
| **EX1** | 1.00 | | | | | | | | | | | | | | |
| **EX2** | .00 | 1.00 | | | | | | | | | | | | | |
| **EX3** | .08 | .05 | 1.00 | | | | | | | | | | | | |
| **EX4** | .12 | .15 | .28 | 1.00 | | | | | | | | | | | |
| **EX5** | -.01 | .02 | .15 | .05 | 1.00 | | | | | | | | | | |
| **EX6** | .04 | .06 | .02 | .01 | .14 | 1.00 | | | | | | | | | |
| **MD1** | -.14 | -.07 | -.23 | -.16 | -.06 | -.06 | 1.00 | | | | | | | | |
| **MD3** | -.07 | -.01 | -.19 | -.13 | -.03 | -.12 | .46 | 1.00 | | | | | | | |
| **MD5** | -.16 | -.13 | -.21 | -.22 | -.12 | -.17 | .36 | .28 | 1.00 | | | | | | |
| **CI1** | -.15 | -.12 | -.14 | -.23 | -.12 | -.08 | -.19 | -.21 | -.16 | 1.00 | | | | | |
| **CI4** | -.18 | -.18 | -.14 | -.26 | -.10 | -.01 | -.18 | -.25 | -.19 | .48 | 1.00 | | | | |
| **CI5** | -.15 | -.18 | -.18 | -.21 | -.18 | -.15 | -.24 | -.26 | -.16 | .24 | .28 | 1.00 | | | |
| **EI1** | -.15 | -.18 | -.19 | -.18 | -.31 | -.18 | -.21 | -.17 | -.12 | -.05 | -.01 | .10 | 1.00 | | |
| **EI2** | -.13 | -.15 | -.22 | -.18 | -.27 | -.17 | -.18 | -.16 | -.12 | -.05 | -.03 | .08 | .44 | 1.00 | |
| **EI5** | -.13 | -.22 | -.19 | -.14 | -.22 | -.19 | -.20 | -.22 | -.11 | -.09 | -.06 | .09 | .42 | .38 | 1.00 |

**Table S9. Analysis name BAT14 containing 14 items, representative sample of working population NL and FL subsample 2 n=800.**

| Item | EX1 | EX2 | EX3 | EX4 | EX5 | MD1 | MD3 | MD5 | CI1 | CI4 | CI5 | EI1 | EI2 | EI5 |
|---|---|---|---|---|---|---|---|---|---|---|---|---|---|---|
| **EX1** | 1.00 | | | | | | | | | | | | | |
| **EX2** | .01 | 1.00 | | | | | | | | | | | | |
| **EX3** | .09 | .06 | 1.00 | | | | | | | | | | | |
| **EX4** | .13 | .16 | .28 | 1.00 | | | | | | | | | | |
| **EX5** | .01 | .04 | .16 | .06 | 1.00 | | | | | | | | | |
| **MD1** | -.13 | -.07 | -.23 | -.15 | -.06 | 1.00 | | | | | | | | |
| **MD3** | -.07 | -.01 | -.19 | -.13 | -.02 | .46 | 1.00 | | | | | | | |
| **MD5** | -.16 | -.14 | -.22 | -.23 | -.11 | .35 | .27 | 1.00 | | | | | | |
| **CI1** | -.14 | -.11 | -.13 | -.23 | -.11 | -.19 | -.22 | -.18 | 1.00 | | | | | |
| **CI4** | -.18 | -.17 | -.13 | -.26 | -.09 | -.19 | -.26 | -.20 | .48 | 1.00 | | | | |
| **CI5** | -.16 | -.18 | -.18 | -.22 | -.18 | -.25 | -.28 | -.18 | .23 | .27 | 1.00 | | | |
| **EI1** | -.16 | -.19 | -.20 | -.19 | -.31 | -.22 | -.19 | -.14 | -.07 | -.02 | .09 | 1.00 | | |
| **EI2** | -.14 | -.16 | -.23 | -.19 | -.27 | -.20 | -.18 | -.14 | -.07 | -.04 | .07 | .43 | 1.00 | |
| **EI5** | -.14 | -.22 | -.20 | -.15 | -.22 | -.22 | -.24 | -.14 | -.11 | -.07 | .08 | .42 | .37 | 1.00 |

**Table S10. Analysis name BAT13 containing 13 items, representative sample of working population NL and FL subsample 2 n=800.**

| Item | EX1 | EX3 | EX4 | EX5 | MD1 | MD3 | MD5 | CI1 | CI4 | CI5 | EI1 | EI2 | EI5 |
|---|---|---|---|---|---|---|---|---|---|---|---|---|---|
| **EX1** | 1.00 | | | | | | | | | | | | |
| **EX3** | .10 | 1.00 | | | | | | | | | | | |
| **EX4** | .14 | .30 | 1.00 | | | | | | | | | | |
| **EX5** | .02 | .17 | .07 | 1.00 | | | | | | | | | |
| **MD1** | -.13 | -.22 | -.14 | -.05 | 1.00 | | | | | | | | |
| **MD3** | -.06 | -.18 | -.11 | -.01 | .46 | 1.00 | | | | | | | |
| **MD5** | -.17 | -.22 | -.22 | -.12 | .34 | .27 | 1.00 | | | | | | |
| **CI1** | -.15 | -.13 | -.22 | -.12 | -.20 | -.23 | -.19 | 1.00 | | | | | |
| **CI4** | -.19 | -.14 | -.25 | -.10 | -.20 | -.27 | -.22 | .47 | 1.00 | | | | |
| **CI5** | -.17 | -.19 | -.22 | -.19 | -.27 | -.29 | -.20 | .22 | .26 | 1.00 | | | |
| **EI1** | -.17 | -.21 | -.18 | -.32 | -.24 | -.20 | -.16 | -.08 | -.04 | .07 | 1.00 | | |
| **EI2** | -.14 | -.23 | -.18 | -.28 | -.21 | -.19 | -.16 | -.08 | -.06 | .05 | .42 | 1.00 | |
| **EI5** | -.15 | -.20 | -.15 | -.23 | -.24 | -.26 | -.16 | -.13 | -.09 | .06 | .41 | .36 | 1.00 |

**Table S11. Analysis name BAT12 containing 12 items, representative sample of working population NL and FL subsample 2 n=800.**

| Item | EX1 | EX3 | EX4 | MD1 | MD3 | MD5 | CI1 | CI4 | CI5 | EI1 | EI2 | EI5 |
|---|---|---|---|---|---|---|---|---|---|---|---|---|
| **EX1** | 1.00 | | | | | | | | | | | |
| **EX3** | .12 | 1.00 | | | | | | | | | | |
| **EX4** | .16 | .32 | 1.00 | | | | | | | | | |
| **MD1** | -.13 | -.20 | -.13 | 1.00 | | | | | | | | |
| **MD3** | -.05 | -.15 | -.09 | .45 | 1.00 | | | | | | | |
| **MD5** | -.17 | -.21 | -.21 | .34 | .27 | 1.00 | | | | | | |
| **CI1** | -.15 | -.12 | -.22 | -.21 | -.23 | -.21 | 1.00 | | | | | |
| **CI4** | -.19 | -.13 | -.25 | -.20 | -.27 | -.23 | .47 | 1.00 | | | | |
| **CI5** | -.18 | -.19 | -.23 | -.29 | -.30 | -.22 | .20 | .25 | 1.00 | | | |
| **EI1** | -.19 | -.22 | -.20 | -.27 | -.23 | -.20 | -.11 | -.07 | .05 | 1.00 | | |
| **EI2** | -.16 | -.24 | -.19 | -.24 | -.21 | -.19 | -.10 | -.08 | .03 | .40 | 1.00 | |
| **EI5** | -.16 | -.21 | -.16 | -.26 | -.28 | -.19 | -.15 | -.11 | .04 | .38 | .34 | 1.00 |
