# Supplementary material for: Shortening of the Burnout Assessment Tool (BAT)—from 23 to 12 items using content and Rasch analysis
Source: BMC Public Health. 2022 Mar 22;22:560. doi: 10.1186/s12889-022-12946-y (PMC8939057; doi:10.1186/s12889-022-12946-y)
Supplement: Supplementary file 4 — Additional file 4. [file 12889_2022_12946_MOESM4_ESM.pdf]

Appendix 4

Table S1. Location and threshold values, from the BAT15a analysis containing 15 items, subsample 1 n=800.

		Thresholds			
**Item**	**Location**	**1**	**2**	**3**	**4**
EX1	-0.35863	-3.29611	-0.9751	0.867487	1.969211
EX2	-0.10428	-3.45186	-0.43543	0.98581	2.484356
EX3	-0.39893	-3.2474	-1.11062	0.649203	2.113091
EX4	-0.24121	-3.25452	-0.84877	0.64155	2.496924
EX5	-0.66527	-3.48506	-1.39065	0.45007	1.76457
EX6	-0.08915	-3.04256	-0.72514	1.053743	2.357375
MD1	0.050773	-2.30434	-0.45173	0.636208	2.322963
MD3	0.289135	-1.69092	-0.6237	1.078627	2.392531
MD5	-0.13038	-2.31335	-1.08127	0.729542	2.143535
CI1	-0.07283	-3.38582	-0.61469	1.174595	2.534579
CI4	0.01704	-3.55903	-0.59398	1.350976	2.870197
CI5	0.090844	-3.34239	-0.2304	1.782608	2.153558
EI1	0.69135	-1.63402	-0.04962	1.446779	3.002269
EI2	0.523937	-1.54868	-0.28295	1.096485	2.830891
EI5	0.397598	-2.0673	-0.52331	1.072071	3.10893

Table S2. Location and threshold values, from the BAT15b analysis containing 15 items, subsample 1 n=800.

		Thresholds			
**Item**	**Location**	**1**	**2**	**3**	**4**
EX1	-0.31593	-3.25314	-0.93159	0.916954	2.004061
EX2	-0.05801	-3.40168	-0.38849	1.020941	2.537192
EX3	-0.35175	-3.20318	-1.06987	0.692816	2.173223
EX4	-0.19856	-3.21025	-0.80414	0.686387	2.533781
EX5	-0.62163	-3.4413	-1.34868	0.495496	1.807965
EX6	-0.04862	-2.99269	-0.67896	1.091249	2.385939
MD1	0.090835	-2.25664	-0.40314	0.676134	2.346986
MD3	0.329392	-1.64383	-0.58034	1.116881	2.424853
MD5	-0.08746	-2.27084	-1.03625	0.775354	2.181903
CI1	-0.03326	-3.33717	-0.5687	1.213949	2.55888
CI4	0.059259	-3.51675	-0.5457	1.383544	2.915946
CI5	0.131637	-3.29734	-0.18337	1.80937	2.197893
EI1	0.726964	-1.5753	-0.01273	1.467687	3.028197
EI2	0.560074	-1.48917	-0.24799	1.122529	2.854927
EI3	-0.18296	-3.12336	-1.29429	0.68532	3.000514

Table S3. Location and threshold values, from the BAT13 analysis containing 13 items, subsample 1 n=800.

		Thresholds			
**Item**	**Location**	**1**	**2**	**3**	**4**
EX1	-0.41874	-3.33934	-1.02239	0.807846	1.878904
EX3	-0.17138	-3.4918	-0.48512	0.91581	2.375611
EX4	-0.46136	-3.27117	-1.16049	0.585756	2.000462
EX6	-0.30463	-3.27891	-0.90511	0.581989	2.383518
MD1	-0.01311	-2.35277	-0.50312	0.575317	2.228148
MD3	0.228734	-1.73858	-0.67865	1.020582	2.311581
MD5	-0.18967	-2.36269	-1.12579	0.672541	2.057279
CI1	-0.13001	-3.44375	-0.66619	1.124071	2.46585
CI4	-0.0443	-3.61424	-0.64254	1.292117	2.787478
CI5	0.044854	-3.39113	-0.28609	1.726988	2.12965
EI1	0.636152	-1.68897	-0.09126	1.394446	2.930393
EI2	0.479956	-1.60761	-0.32443	1.049143	2.802723
EI5	0.343484	-2.12863	-0.56614	1.031192	3.037508
